# Glial Tiling in the Insect Nervous System

**DOI:** 10.3389/fncel.2022.825695

**Published:** 2022-02-17

**Authors:** Nicole Pogodalla, Bente Winkler, Christian Klämbt

**Affiliations:** Institute for Neuro- and Behavioral Biology, University of Münster, Münster, Germany

**Keywords:** tiling, glia, Drosophila, central nervous system, cell-cell contacts

## Abstract

The Drosophila nervous system comprises a small number of well characterized glial cell classes. The outer surface of the central nervous system (CNS) is protected by a glial derived blood-brain barrier generated by perineurial and subperineurial glia. All neural stem cells and all neurons are engulfed by cortex glial cells. The inner neuropil region, that harbors all synapses and dendrites, is covered by ensheathing glia and infiltrated by astrocyte-like glial cells. All these glial cells show a tiled organization with an often remarkable plasticity where glial cells of one cell type invade the territory of the neighboring glial cell type upon its ablation. Here, we summarize the different glial tiling patterns and based on the different modes of cell-cell contacts we hypothesize that different molecular mechanisms underlie tiling of the different glial cell types.

## Introduction

There is no doubt that a complex nervous system requires a precise and reproducible arrangement of its cellular components, neurons and all non-neuronal cells. During development, all neural cells must find their appropriate neighbors to correctly intermingle. In most cases, neurons innervate their targets in a non-redundant or non-overlapping manner, which is called tiling. Such tiling patterns provide a maximal coverage of a given area with little overlap at cell boundaries and thus tiling minimizes the number of cells needed to fulfill a specific task and may facilitate the efficient organization of precise cartographic maps in the developing nervous system.

During nervous system development tiling principles first govern the regular distribution of progenitor cells such as the radial glial cells (Taverna et al., 2014). Here, a regulator of microtubule stability, Memo1, is needed to establish a regularly spaced pattern of these progenitor cells (Nakagawa et al., 2019). Later during development such mechanisms are needed to ensure a tile like arrangement of specific neurons with minimal overlap to avoid intercellular competition. In fly sensory neurons, this is brought about by the seven-pass transmembrane atypical cadherin protein Starry night (Flamingo) that is also involved in planar cell polarity (Grueber et al., 2002; Chen et al., 2008). In mammals, such tiling processes are frequently observed, too. For example, regularly spaced neurons of the hippocampus and the entorhinal complex, place and grid cells, provide spatial representation of the environment and thus allow navigation of the animal. The cartographic representation is defined by complex neuronal activities, but how the underlying regular distribution of neurons is controlled is not known yet (O’Keefe and Dostrovsky, 1971; Rowland et al., 2016; Rueckemann et al., 2021).

In contrast, the molecular mechanisms that are in place to ensure tiling on a single cell level, and thus an isotypic level, are being unraveled. First identified in Drosophila but later also shown in mammals, neurons express a specific combination of the Ig-domain protein DSCAM which ensures that all cell processes carrying the same DSCAM variants repel each other (Hattori et al., 2007, 2008; Schmucker, 2007; Lefebvre et al., 2015; Guo et al., 2021). Thereby intracellular competition is reduced or avoided. In addition, the Ig-domain protein Turtle (Tutl) is cell-autonomously needed to ensure dendrite self-avoidance in neurons with complex dendritic morphologies (Long et al., 2009). Interestingly, the cytoplasmic tail of Tutl is dispensable for control of dendrite branching, suggesting that Tutl either acts as a membrane-bound signaling ligand or as a co-receptor in a larger receptor complex (Long et al., 2009). Turtle is also expressed by Drosophila photoreceptor cells and is known to interact with the Ig-domain Borderless which is expressed by wrapping glial cells located in the optic nerve. Here, this interaction is needed to regulate proper axonal ensheathment (Cameron et al., 2016; Chen et al., 2017). Possibly, the Turtle - Borderless signaling pair also participates in the regular tiled positioning of wrapping glial cells that has been found in the optic nerve (Silies et al., 2007; Franzdóttir et al., 2009).

## Tiling of Mammalian Glial Cells

Tiling of glial cells has been intensively documented for the mammalian nervous system. Microglial cells are the immune competent cells of the brain. They originate from the yolk sack during very early embryonic development and invade the brain. Here, they are positioned in a tiled pattern waiting during the surveillance of the immune status of the brain (Eyo et al., 2018). Unfortunately, not much is currently known on the molecules that direct the regular spacing of these cells.

Macroglia of the central nervous system (CNS) are either oligodendrocytes or astrocytes, which both originate from the neural tube. Astrocytes are known for an extensive tiling in the functioning nervous system (Khakh and Deneen, 2019). Recently, first molecules were identified that provide an entry into dissecting molecular mechanisms regulating underlying astrocyte-astrocyte interactions (Baldwin et al., 2021). The Ig-domain cell adhesion molecule HepaCAM, also known as GlialCAM, can homophilically interact and is known to modulate membrane trafficking and thus the composition of the plasma membrane (Barrallo-Gimeno and Estévez, 2014; Bosch and Estévez, 2020). Thereby HepaCAM regulates astrocyte morphology and positioning in the developing mouse brain (Baldwin et al., 2021).

The HepaCAM protein harbors two Ig-domains that show highest homology to Ig-domains of several Drosophila proteins: DSCAM4 (*p* = 5.5e-08), Fas2 (*p* = 4e-06) and Sdk (*p* = 5.7e-05). All three fly proteins are known adhesion proteins (Tadros et al., 2016; Letizia et al., 2019; Neuert et al., 2020), and all three genes show at least some expression in adult glial cells (Davie et al., 2018) – but whether these proteins contribute in tiling processes remains to be addressed. In addition, Drosophila harbors two Ig-domain proteins with the same molecular signature (Ig, Igc2, transmembrane domain, cytoplasmic domain), Basigin, and Sidestep but their contribution to tiling is not yet addressed either.

Possibly, the tiling principle also provides a mechanism to cope with loss of cells due to neurodegeneration or disease. When, for example in invertebrates, single astrocytes are lost in the nervous system, neighboring astrocytes are able to invade the free space and ensure that phenotypic consequences in respect to neuronal functionality are kept low (Stork et al., 2014). Thus, the considerable plasticity in the nervous system combined with mechanisms ensuring tiled organization of neurons and glia provide the means to efficiently repair small lesions in the nervous system.

## Tiling of Drosophila Surface Glial Cells

**FIGURE 1:**
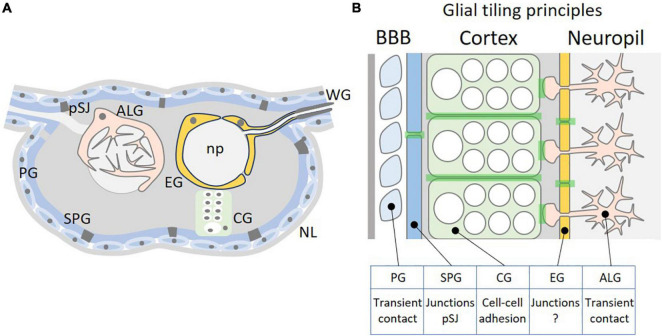
Tiling of glial cells of the Drosophila CNS. **(A)** Schematic cross-section through the Drosophila CNS. The outer surface of the nervous system is covered by a thick extracellular matrix called neural lamella (NL). Below are the perineurial and subperineurial glial cells (PG and SPG) that establish the blood-brain barrier by forming pleated septate junctions (pSJ). Neural stem cells and neuronal cell bodies are covered by the cortex glia (CG). The inner neuropil (np) is covered by astrocyte-like glial and ensheathing glial cells. For a schematic view of the adult CNS please see Kremer et al. (2017). **(B)** Possible tiling principles among different glial cell classes. PGs have only transient contact, whereas SPGs are polarized cells that establish extensive pleated septate junctions. CGs interact along a large cellular interface and tiling likely involves adhesion. EGs are polarized cells and form a barrier similar as the SPGs. However, no specific cell-cell junctions have been clearly documented between EGs. ALGs tile the neuropil without forming extensive cell-cell contacts and therefore tiling is likely to require transient contacts, too. The light green shading indicates cell-cell interaction zones where tiling principles are acting.

Tiling is a fundamental organizational principle to reduce the number of cells needed during development and indeed all Drosophila glial cells show some tiling behavior. Within the fly CNS only 10% of all neural cells are of glial nature. They can be classified as surface glia comprising perineurial and subperineurial glia, cortex glia and neuropil-associated glia comprising astrocyte-like and ensheathing glial cells (Figure 1A). A tiling pattern is most obvious example of epi- and endothelial tissues. Such tissue properties are also found in the nervous system where they are utilized to form the blood-brain barrier. In all organisms, the nervous system needs to be protected from circulation by a tight and, most importantly, selective barrier (Carlson et al., 2000; Obermeier et al., 2013). In Drosophila, as in other invertebrates and primitive vertebrates, this barrier is made by glial cells that perform evolutionarily conserved functions (Bundgaard and Abbott, 2008; Mayer et al., 2009; Schirmeier and Klämbt, 2015; Hindle et al., 2017).

The fly blood-brain barrier is formed by two specific cell types, the perineurial and the subperineurial glial cells. These two cell types differ in a number of characteristics, but both tile the nervous system in a very regular manner (Figure 1). The perineurial glial cells form the outer-most cellular layer of the nervous system abutting a dense layer of extracellular matrix called neural lamella. Their function is associated with metabolic homeostasis of the nervous system and thus they do express specific transporters that for example can take up carbohydrates from circulation, the hemolymph (Limmer et al., 2014). The perineurial glia are able to divide throughout their development and cover the entire nervous system [CNS and peripheral nervous system (PNS)] in a complex but regular pattern (Awasaki et al., 2008; Kremer et al., 2017). For the PNS it was suggested that division of perineurial glial cells is controlled by a mechanism that involves contact-inhibition (Matzat et al., 2015; Zülbahar et al., 2018), but the underlying molecules are elusive. Due to missing cell-cell junctions, we anticipate that tiling mechanisms are due to transient cell-cell contacts (Figure 1B).

The subperineurial glial cells are situated between the perineurial glial cells and the CNS cortex. They establish a physical diffusion barrier by the formation of occluding junctions and tile the complete nervous system, whereas the perineurial glia together with the neural lamella only act as a non-specific filter to prevent access of high molecular weight molecules into the brain (Carlson et al., 2000; Stork et al., 2008; Hindle and Bainton, 2014).

The barrier function of subperineurial glial cells is established at the end of embryonic development and starts about 9 h after egg laying (AEL) at 25°C (Stork et al., 2008; Schwabe et al., 2017). In every embryonic hemineuromere only eight subperineurial glial cells are formed. During subsequent embryonic development, the different subperineurial glial cells migrate to the surface of the CNS. As soon as they reach the brain surface they stop and synchronously extend lateral cell processes toward their neighbors to tile the nervous system (Schwabe et al., 2017). The extension of lateral processes requires polarization and contact of the subperineurial glial cells with the neural lamella, which surrounds the entire nervous system (Tepass et al., 1994; Olofsson and Page, 2005). This mesenchymal-epithelial transition is then stabilized by the formation of cell-cell specific occluding junctions, called pleated septate junctions (pSJ; Schwabe et al., 2017).

During early developmental stages subperineurial glial cells are not yet covered by an extensive layer of perineurial glial cells and thus are able to directly interact with the extracellular matrix proteins of the neural lamella using the Dystroglycan (Dg) receptor (Schwabe et al., 2017). Loss of ECM proteins triggered by a genetic ablation of macrophages demonstrates that although subperineurial glial cell migration and polarity is not altered, subsequent isometric growth is disrupted and as consequence the establishment of septate junctions that set up the actual diffusion barrier fails (Schwabe et al., 2017; Babatz et al., 2018). In contrast to perineurial glial cells, subperineurial glial cells do not divide but rather increase in cell size (Awasaki et al., 2008; Stork et al., 2008; Unhavaithaya and Orr-Weaver, 2012). This is in particular dramatic during larval development, when the body size increases up to 100 times. Thus, at the end of larval development, subperineurial glial cells must undergo an enormous growth to cover the expanding nervous system and to ensure a constant barrier function. To achieve this growth, they become polyploid and sometimes polynucleated (Unhavaithaya and Orr-Weaver, 2012; Von Stetina et al., 2018; Zülbahar et al., 2018). Interestingly, the pSJ that are needed to block paracellular diffusion between different subperineurial glial cells are established already during early embryonic stages (Babatz et al., 2018). Based on electron microscopic analysis, they appear to consist of multiple parallel pleated 10–20 nm wide strands (Gilula et al., 1970; Lane and Swales, 1982) generated by a large number of proteins that in contrast to highly dynamic adhesion junctions, are very stable (Babatz et al., 2018; Petri et al., 2019). One important regulator involved in proper pSJ stand is the G protein-coupled receptor Moody (Bainton et al., 2005; Schwabe et al., 2005). *moody* mutants fail to form proper septate junction strands and thus a disrupted BBB is expected. Interestingly, however, this reduced barrier function is rescued by the formation of interdigitating membrane folds and possibly other junctional structures established here (Babatz et al., 2018).

Thus, tiling of the subperineurial glial cells appears to be a direct consequence of the cell- cell junctions expressed. In case of removal of these junctions, neighboring subperineurial glial cells interdigitate to increase the length of the diffusion path (Babatz et al., 2018). How a change in these physiological parameters then changes the extend of cellular overlap remains to be analyzed.

As discussed above, tiling of subperineurial glial cells is well described for *Drosophila melanogaster* and tiling possibly involves junctional contacts such as pleated septate junctions. An open question is whether other insects show similar tiling principles of the cells constituting the blood-brain barrier. Several classic electron microscopic studies suggest that the blood-brain barrier in the stick insect *Carausius morosus*, the cockroach *Periplaneta americana* or the moth *Manduca sexta* is organized in a similar manner as found for Drosophila (Maddrell and Treherne, 1967; Lane et al., 1977; Lane and Swales, 1979). In the classic studies several junctional contact principles were discussed between blood-brain barrier forming cells such as tight junctions, desmosomes, scalarifom-like junctions or septate junctions (Lane, 1991). Recent molecular experiments revealed many of the membrane proteins required to form septate junctions (Petri et al., 2019). One of the septate junction core proteins is Neurexin IV (Oshima and Fehon, 2011). Interestingly, two distinct Neurexin IV isoforms are made by mutual exclusive splicing of either exon three or exon four. Whereas Neurexin IV^*Exon*4^ is expressed only by neurons, Neurexin IV^*Exon*3^ is found specifically in septate junction forming cells (Rodrigues et al., 2012). Recent sequencing projects revealed similar Neurexin IV isoforms in *Manduca*, *Nasonia*, *Tribolium*, *Galleria*, and *Apis*, suggesting that findings made in the Drosophila system might be more generally applicable in insects. However, tiling needs to be further demonstrated by specific cell labeling experiments using antibodies detecting specific cell adhesion proteins or Brainbow-like experiments.

The explicitly tiled blood-brain barrier not only prevents uncontrolled leakage of solutes into and out of the CNS. It also protects the nervous system against invasion of pathogens and immune cells, which in principle can occur *via* a transcellular or a paracellular route. For the mammalian blood-brain barrier passage through tricellular junctions is detected most frequently (Dias et al., 2021). In invertebrates, breaching of an intact blood-brain barrier has been described only recently in response to glial immunity induction (Winkler et al., 2021). However, a preferred passage route into the nervous system is not yet known. Once macrophages have entered the nervous system, they move toward the neuropil, where they start to phagocytose synaptic material (Winkler et al., 2021).

## Tiling of Glial Cells Within the Central Nervous System

When macrophages enter the nervous system, they encounter a well-organized and highly structured cellular environment and at least initially they do not come into contact with neuronal cell processes (Figure 1). In larvae, the first neural cells, which macrophages contact once they have crossed the subperineurial glial cells, are the cortex glial cells that ensheath all neuroblasts and their progeny (Pereanu et al., 2005; Coutinho-Budd et al., 2017). As the subperineurial glial cells, the cortex glial cells tile the CNS, too. Cortex glial cells are very large, honey-combed structured cells that can surround up to 100 neuronal cell bodies and tile the inner cortex of the nervous system except for the very dorsal part of the CNS (Pereanu et al., 2005; Coutinho-Budd et al., 2017; Pogodalla et al., 2021). Thereby they separate the neuronal cell bodies of specific neuronal lineages from glial cells that surround the synaptic compartment called the neuropil: astrocyte-like glial cells and ensheathing glial cells.

Cortex glia and astrocyte-like glia occupy non-overlapping brain volumes and upon cortex glia ablation, astrocyte-like cells invade the CNS cortex whereas cortex glial cells invade the neuropil after astrocyte-like glial cell ablation (Coutinho-Budd et al., 2017). Thus, tiling principles are not restricted to a single cell type but rather appear based on interaction between several subtypes, suggesting that more globally expressed membrane proteins are responsible for such patterning mechanisms (Figure 1B). Candidate proteins might be Cadherin-type adhesion proteins, that show a widespread expression in the nervous system and are required in cortex glia for correct positioning of neuronal cell bodies and neuroblasts (Dumstrei et al., 2003; Fung et al., 2008).

Invading macrophages would therefore have to migrate through a cortex glial cell to directly contact neuronal cell membranes or they could navigate along the cortex glial cell boundaries toward the neuropil. Here, they encounter another barrier that is formed by the neuropil glia. Two different neuropil-associated glial cells are known, astrocyte-like glial and ensheathing glial cells. In every hemineuromer, six astrocyte-like glial cells and three ensheathing glial cells originate from a common precursor cell, known as the longitudinal glioblast (Beckervordersandforth et al., 2008; Omoto et al., 2015; Peco et al., 2016). The differentiation of the two neuropil-associated glial cell types is determined by Notch and EGF-receptor signaling (Peco et al., 2016).

In larvae, the six astrocyte-like glial cells are found at the dorsal part of the neuropil. They send a main process into the neuropil where it branches out and generates a dense meshwork of very fine processes that covers a specific and unique synaptic volume (Stork et al., 2014; MacNamee et al., 2016; Peco et al., 2016; Kremer et al., 2017). Similar as mammalian astrocytes, Drosophila astrocytes tile the synaptic neuropil and do not invade the territories occupied by neighboring astrocytes, where local interactions are needed during neural circuit assembly (Stork et al., 2014; Peco et al., 2016; Ackerman et al., 2021; Figure 1). Although this tiling behavior has been beautifully documented, we still do not understand the underlying molecular mechanisms.

Within a given synaptic volume astrocyte-like cells are regulating synaptic activity and possibly are able to coordinate neuronal activity *via* Ca^2+^ waves (Panatier et al., 2011; Zhang et al., 2017; Weiss et al., 2022). In Drosophila, astrocyte-like glial cells are coupled by gap junctions and demonstrate robust spontaneous Ca^2+^ oscillatory activity, comparable to local Ca^2+^ transients observed in cortex glia. An acute astrocyte Ca^2+^ influx leads to rapid endocytosis of GABA transporter (Gat) causing a disrupted GABA uptake which results in suppression of neuronal activity and paralysis (Ma et al., 2016; MacNamee et al., 2016; Zhang et al., 2017; Ma and Freeman, 2020).

Three ensheathing glia are generated by the embryonic longitudinal glioblast, while one additional ensheathing glial cell is formed by a separate lateral neuroblast. In the early larval ventral nerve cord, the four ensheathing glial cells are positioned in a stereotyped pattern around the neuropil. They can be placed into two distinct morphological classes. Within one larval hemineuromer, two ventrally localized ensheathing glial cells tile the surface of the ventrolateral neuropil. In addition, two so-called ensheathing/wrapping glial cells are found in each hemineuromer. This subtype participates in tiling mostly of the dorsal neuropil surface and, in addition, covers the nerve roots that harbor the sensory axons entering the CNS and the motor axons that leave the CNS towards the muscles (Pogodalla et al., 2021). As the larva matures, these cells grow in size and extend lateral processes to fully cover the neuropil. This forms an internal barrier-like structure separating the CNS cortex from the neuropil (Figure 1). Indeed, dye injection experiments into control third instar larvae as well as in third instar larvae lacking ensheathing glia have shown that the ensheathing glial cells establish such an internal barrier around the neuropil (Pogodalla et al., 2021). Moreover, ablation of the ensheathing glia resulted in compensatory growth of astrocyte-like glia similar to what has been noted upon cortex glia ablation (Coutinho-Budd et al., 2017; Pogodalla et al., 2021). Upon ensheathing glia ablation, astrocyte-like glial cells extend processes around the neuropil again highlighting glial plasticity to ensure a robust development and function of the CNS.

A barrier formed by tiled cells suggests that these cells are polarized as in the blood-brain barrier or in all epithelial barriers. Indeed, further cell biological characterization has demonstrated a clear cellular polarity of the ensheathing glia with an apical plasma membrane domain facing the neuropil (Pogodalla et al., 2021). Thus, given the cell polarity of the ensheathing glial cells, cell-cell junctions may serve as molecular mechanism to ensure tiling. In contrast to what we have seen for subperineurial glial cells, no septate junction-like structures can be identified between different ensheathing glial cells in electron microscopic images. However, some reports have demonstrated the presence of scalariform-like junctions between specific neuropil glial cells in moths as well as in Drosophila (Tolbert and Hildebrand, 1981; Oland et al., 2008). Scalariform-like junctions are characterized by fine tubular pillars appearing in cross sections of electron microscopy images as a ladder-like structure with a regular extracellular space of 20 nm width (Noirot-Timothée and Noirot, 1980). These junctions might stabilize the cell-cell contacts made by the tiled ensheathing glia to ensure a stable internal barrier. In addition to possibly establishing some stabilizing junctions, ensheathing glial cell processes continue to extend lateral processes leading to overlapping cell-cell contacts, which increase the diffusion path and thus contribute to the establishment of the barrier function.

## Tiling of Glial Cells Within the Peripheral Nervous System

The PNS in Drosophila is covered by a blood-brain barrier that is made by the same cellular components as the blood-brain barrier of the CNS (Stork et al., 2008). Moreover, extensive searches for enhancer elements specific to either CNS or PNS blood-brain barrier failed suggesting that very similar molecular mechanisms operate in the blood-brain barrier of CNS and PNS. In addition to the blood-brain barrier forming glia, peripheral nerves only harbor the wrapping glia, which is considered to be a Remak-like Schwann cell type equivalent in Drosophila (Nave and Werner, 2021). Only very few wrapping glial cells are found along the segmental nerves of the Drosophila larva which are organized in a tiled fashion (von Hilchen et al., 2013; Matzat et al., 2015). During development, embryonic peripheral glial cells migrate in a collective manner along pre-existing axon trajectories in part guided by repulsive Netrin signaling as well as Notch signaling (Edenfeld et al., 2007; von Hilchen et al., 2010). In the Drosophila wing, collective glial cell migration is detected along sensory nerves (Aigouy et al., 2004). Cell ablation experiments have demonstrated the necessity of cell-cell contacts during glial migration and thus positioning of the glial cells along the nerve (Aigouy et al., 2008). The notion that cell-adhesion is necessary for glial migration is corroborated by the finding that levels of N-Cadherin are involved in controlling glial migration (Kumar et al., 2015). It would be therefore interesting to investigate the impact of N-Cadherin on the tiling behavior along the sensory wing nerve.

In summary, glial tiling is a conserved and critical process within the nervous system to ensure proper brain function as well as minimizing the number of needed cells in an efficient manner. However, our understanding about the mechanisms controlling glial tiling is far from being complete and further investigations are needed to dismantle whether glial tiling also exists in other insects and whether this will be based on evolutionary conserved mechanistic principles.

## Author Contributions

All authors listed have made a substantial, direct, and intellectual contribution to the work, and approved it for publication.

## Conflict of Interest

The authors declare that the research was conducted in the absence of any commercial or financial relationships that could be construed as a potential conflict of interest.

## Publisher’s Note

All claims expressed in this article are solely those of the authors and do not necessarily represent those of their affiliated organizations, or those of the publisher, the editors and the reviewers. Any product that may be evaluated in this article, or claim that may be made by its manufacturer, is not guaranteed or endorsed by the publisher.
